# Time course of eosinophilic myocarditis visualized by CMR

**DOI:** 10.1186/1532-429X-10-21

**Published:** 2008-05-08

**Authors:** Kurt Deb, Behrus Djavidani, Stefan Buchner, Florian Poschenrieder, Norbert Heinicke, Stefan Feuerbach, Günter Riegger, Andreas Luchner

**Affiliations:** 1 Klinik und Poliklinik für Innere Medizin II, Klinikum der Universität, Regensburg, Germany; 2 Institut für Röntgendiagnostik, Klinikum der Universität, Regensburg, Germany

## Abstract

We report the diagnostic potential of cardiovascular magnetic resonance (CMR) to visualize the time course of eosinophilic myocarditis upon successful treatment. A 50-year-old man was admitted with a progressive heart failure. Endomyocardial biopsies were taken from the left ventricle because of a white blood cell count of 17000/mm^3 ^with 41% eosinophils. Histological evaluation revealed endomyocardial eosinophilic infiltration and areas of myocyte necrosis. The patient was diagnosed with hypereosinophilic myocarditis due to idiopathic hypereosinophilic syndrome. CMR-studies at presentation and a follow-up study 3 weeks later showed diffuse subendocardial LGE in the whole left ventricle. Upon treatment with steroids, CMR-studies revealed marked reduction of subendocardial LGE after 3 months in parallel with further clinical improvement. This case therefore highlights the clinical importance of CMR to visualize the extent of endomyocardial involvement in the diagnosis and treatment of eosinophilic myocarditis.

## Case report

**Figure 1:**
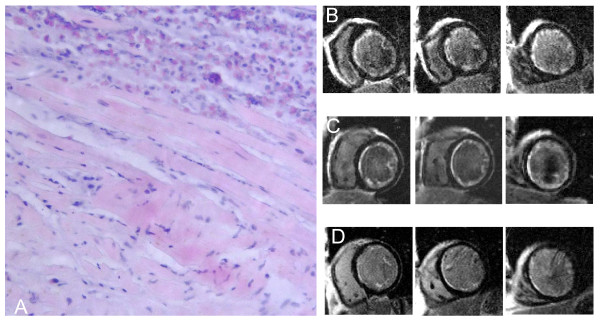
(A) Endomyocardial biopsy specimen. Extensive eosinophilic infiltrate involving the endocardium and myocardium (hematoxylin and eosin). Corresponding CMR short-axis slices (basal, middle, apical) during acute presentation (B), after 3 weeks (C), and after 3 months (D) showing marked regression of subendocardial LGE.

A 50-year-old man was admitted with a suspicion of an acute coronary syndrome because of progressive dyspnea and positive Troponin I (9.5 ng/ml). A two-dimensional echocardiogram revealed severe left ventricular hypokinesis with an ejection fraction of 27%. Upon coronary angiography, coronary artery disease was excluded. Because of a white blood cell count of 17000/mm^3 ^with 41% eosinophils, endomyocardial biopsies were taken from the left ventricle. Histological evaluation showed marked endomyocardial eosinophilic infiltration and areas of myocyte necrosis (Figure 1A). Further evaluation revealed no evidence of secondary hypereosinophilia (malignant diseases, allergy, vasculitis, parasitic infection). The patient was diagnosed with hypereosinophilic myocarditis due to idiopathic hypereosinophilic syndrome. Medication with steroids and heart failure was initiated promptly and the patient improved rapidly.

CMR-studies at presentation and a follow-up study 3 weeks later showed diffuse subendocardial LGE in the whole left ventricle with involvement of the papillary muscles. Upon 3 months follow up, however, subendocardial LGE has markedly decreased in parallel with further clinical improvement (Figures 1B,C,D). Ejection fraction has improved from 27% at baseline to 35% after 3 months and end diastolic volumes have decreased from 195 ml to 161 ml. There was no evidence of mural thrombi at baseline and during follow-up studies and there were no signs of restrictive filling patterns in Doppler and tissue-Doppler echocardiography. NT-pro-BNP decreased from initially 16319 pg/ml to 5305 pg/ml at 3 weeks and to 1926 pg/ml at 3 months.

In conclusion, diagnosis of eosinophilic myocarditis due to idiopathic hypereosinophilic syndrome was made in the early stage. Upon treatment with steroids, CMR-studies revealed marked reduction of subendocardial LGE representing acute inflammation and necrosis. Treatment with steroids in the early stage might have prevented further progression to the intermediate thrombotic-necrotic stage with mural thrombi and the fibrotic stage which has been postulated as the final stage in the time course of eosinophilic myocarditis [1-4].

This case highlights the clinical importance of CMR which is the only noninvasive method to visualize the extent of endomyocardial involvement in the diagnosis and treatment of eosinophilic myocarditis.
